# Stress and Reproductive Hormones of Free-Ranging Dolphins Across a Natural Salinity Gradient

**DOI:** 10.1021/acsomega.4c05466

**Published:** 2024-10-29

**Authors:** Makayla A. Guinn, Justin Y. Elliott, Christiana S. Wittmaack, Carrie Sinclair, Hussain A. Abdulla, Dara N. Orbach

**Affiliations:** † Texas A&M University-Corpus Christi, 6300 Ocean Drive, Corpus Christi, Texas 78412, United States; ‡ Hydrosphere, 2034 Beacon Avenue, Monroe, North Caerolina 28110, United States; § National Oceanic and Atmospheric Administration, Southeast Fisheries Science Center, Mississippi Laboratories, 3209 Frederic Street, Pascagoula, Mississippi 39567, United States

## Abstract

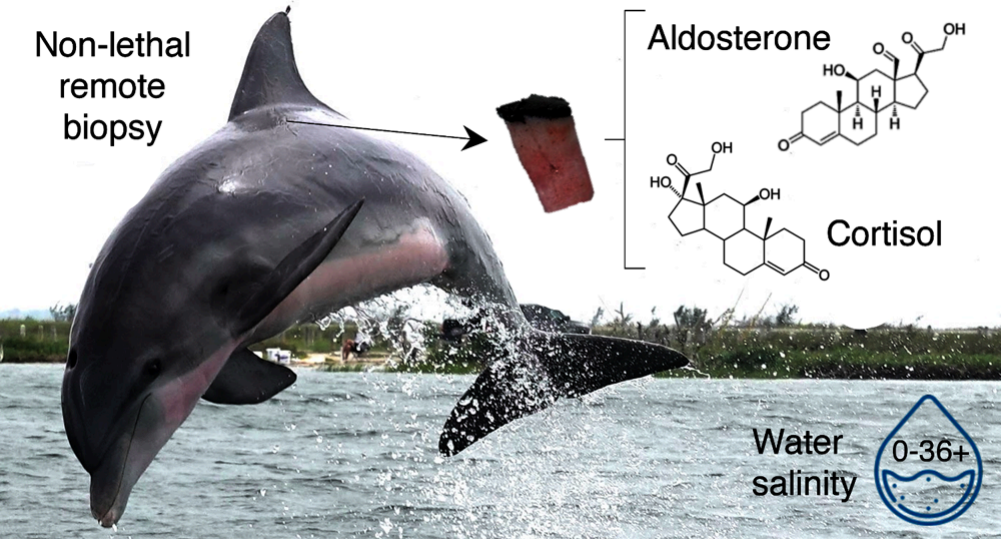

Bottlenose dolphins (*Tursiops truncatus*) inhabit environments with variable natural salinities and experience physiological imbalances from extreme salinity fluctuations. Low salinity exposure (≤10) disrupts osmoregulation and increases the production of steroid hormones aldosterone (electrolyte balance) and cortisol (stress response). The effect(s) of high salinity exposure (>35) on steroid hormone production in bottlenose dolphins has not previously been assessed. We measured stress hormones (aldosterone, corticosterone, cortisol, and cortisone) and reproductive hormones (progesterone and testosterone) in three stocks of free-ranging bottlenose dolphins inhabiting different natural salinities across the Gulf of Mexico (0–30, 22–35, 36+). Hormones were extracted from the blubber of 82 remotely biopsied dolphins and quantified using ultraperformance liquid chromatography coupled with Orbitrap Fusion mass spectrometry. A positive correlation was found between cortisol (10.38 ± 0.8 ng/g, *n* = 13) and salinity (*r* = 0.62). Aldosterone (58.9 ± 17.2 ng/g, *n* = 5) was quantified in dolphin blubber for the first time and was detected across the salinity gradient but was not significantly related to salinity levels. Concentrations of testosterone were seasonally variable. This study enhances our understanding of how climate changes and extensive anthropogenic stressors challenge homeostasis in a marine bioindicator species.

## Introduction

Bay, sound, and estuarine (BSE) stocks of bottlenose dolphins (*Tursiops truncatus*) in the Gulf of Mexico (GoM) can inhabit ecosystems with variable natural salinities.^1,2^ Fluctuations in salinities can be exacerbated by large-scale climate change events that alter global hydrological cycles.^3^ For instance, freshwater inundations from hurricanes can rapidly reduce salinities to low levels that may persist for months.^4^ Conversely, La Niña events create hot and dry climates that can reduce precipitation and raise ambient salinity levels.^5,6^ The average salinity of seawater is 35, although dolphins may inhabit ecosystems with hypo- (<35) and hypersaline (>35) conditions.^7−11^ Low salinity is a physiological stressor in dolphins linked to adverse health conditions (e.g., epidermal lesions, electrolyte imbalances, and mortalities).^12^ Of the six hypersaline lagoons identified globally,^13^ only the Laguna Madre in South Texas has an actively studied stock of dolphins.^11^ Steroid hormones in bottlenose dolphins inhabiting natural hypersaline waters have yet to be assessed, and the effects of high salinity fluxes on dolphin homeostasis are poorly understood. As human impacts continue to drive global climate change and intensify salinity extremes, it is essential to understand the mechanisms that allow an apex bioindicator species, such as the bottlenose dolphin, to maintain physiological balance.

Fluctuations in ambient salinity can induce electrolyte imbalances in dolphins that stimulate the release of aldosterone, a mineralocorticoid hormone, into circulation (i.e., blood and plasma).^14,15^ Circulating aldosterone in bottlenose dolphins can increase above baseline levels simultaneously with cortisol, the mammalian stress hormone, during acute stress events. For instance, prolonged exposure to low salinity,^16,17^ cold water,^18^ and handling by humans^19−21^ can stimulate the dual production of aldosterone and cortisol. Other corticosteroid hormones like cortisone and corticosterone are positively correlated with cortisol and aldosterone,^22,23^ demonstrating a pathway of cascading synthesis during stress response. Seasonal corticosteroid fluxes may be linked to changing environmental conditions and often reflect annual reproductive events.^23^

Like corticosteroids, reproductive hormones such as progesterone and testosterone may be adversely impacted by environmental disturbance.^24,25^ For instance, exposure to lead and pesticide pollutants disrupted progesterone and testosterone synthesis in Indo–Pacific humpback dolphins (*Sousa chinensis*)^26^ and bottlenose dolphins.^27^ The bioavailability of pollutants is directly linked to environmental parameters like salinity,^28^ and many pollutants act as endocrine disruptors that mimic or interfere with normal hormone signaling.^29^ The lipophilic properties of steroid hormones and many pollutants facilitate their sequestration into the lipid-rich blubber of dolphins^30^ where they can accumulate and be metabolized. Hormonal uptake from circulation into the blubber occurs on the scale of several hours to days,^31^ enabling retrospective assessments of the effects of natural and anthropogenic stressors.^23^ As a result, bottlenose dolphins are often used as bioindicators of environmental disturbance.^32−35^ Since hormone transfer from circulation to the blubber is delayed, remote biopsy sampling can be used to collect biological tissue from free-ranging dolphins to assess hormone profiles in free-ranging stocks that do not reflect increased stress from sampling procedures.^31^

With climate change intensifying salinity extremes,^36,37^ there is an increasing need to understand how dolphins regulate major physiological processes in highly variable environments. Blubber is the only matrix in which aldosterone has not yet been quantified in bottlenose dolphins,^31,38^ limiting our knowledge of how aldosterone regulates osmotic balance. To explore the endocrine response of dolphins to variable salinities, we measured stress and reproductive hormones in dolphin blubber across a salinity gradient spanning the northwestern GoM. Hormones were assessed relative to the salinity level, water temperature, dolphin sex, season, and year. We predicted that corticosteroids (cortisol, corticosterone, and cortisone) and aldosterone would be positively correlated with high and low salinities due to increased osmotic pressures.^17^ We did not predict a relationship between salinity and reproductive hormones but did predict that reproductive hormones would vary seasonally.^39^ For samples collected from the same dolphin stock across years, we predicted that stress hormone levels would increase over time due to expanding industrial development and human activity.^40^

## Materials and Methods

### Sampling Locations and Stock Descriptions

**Figure 1:**
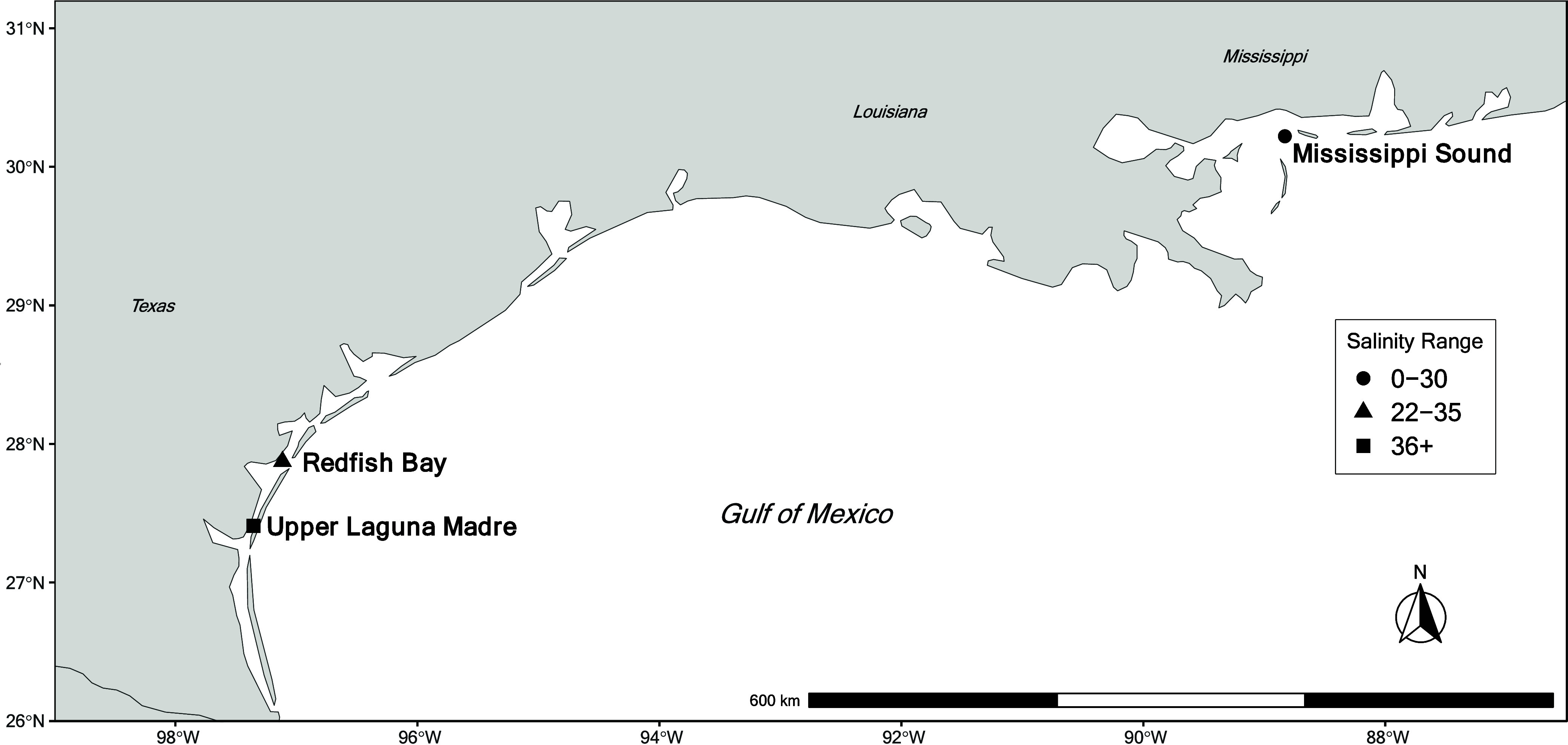
Map of the Northern Gulf of Mexico indicating the three study sites: Mississippi Sound, Redfish Bay, and Upper Laguna Madre. Symbols denote the average salinity range for each sound or bay system: 0–30 (Mississippi Sound), 22–35 (Redfish Bay), and 36+ (Upper Laguna Madre). Modified and reproduced with permission from M. A. Guinn, Sustainability; published by MDPI AG, 2024.

Western Mississippi Sound (MS), Mississippi (Figure 1), experiences large fluctuations in salinities (0–30) and water temperatures (9–33 °C) that are compounded by multiple freshwater river discharges (Mississippi, Pearl, Pascagoula, and Mobile Rivers) and frequent tropical weather.^41^ The Deepwater Horizon oil spill of 2010 exposed resident dolphins to traces of heavy oiling through early 2013.^42−44^ Redfish Bay (RB), Texas (Figure 1), fluctuates seasonally in salinities (22–35) and water temperatures (15–30 °C).^45^ RB is proximate to the largest U.S. port in total revenue tonnage,^46^ >600 mineral production sites, and two superfund sites that have contributed to oil and heavy metal contamination of the bay.^45,46^ Upper Laguna Madre (ULM), Texas (Figure 1), is approximately 100 km southwest of RB and is one of six hypersaline lagoons (>35) occurring globally with salinities regularly exceeding 45.^13,47^ Like MS and RB, the ULM stock of bottlenose dolphins inhabits an area once subjected to high levels of chemical and heavy metal pollution.^45^ All three bottlenose dolphin stocks are considered highly vulnerable to climate change and a high management priority by the National Oceanic and Atmospheric Administration (NOAA).^48^ Proactive monitoring of all three dolphin stocks is warranted to understand the scope of risk posed by anthropogenic contamination of this bioindicator species.

### Remote Biopsy and Photography

Blubber samples were opportunistically collected from bottlenose dolphins from a research boat using a remote biopsy. Prior to sampling, demographics, such as approximate age, dolphin sex, or relationship between individuals in a group, were unknown. Remote biopsy sampling is a less invasive method to collect biological tissue from free-ranging dolphins than direct handling and elicits a minimal behavioral response, with strong evidence for rapid epidermal regeneration and wound healing.^49−53^ Dolphin groups (individuals within 10 m of one another engaged in the same predominant behavioral state^54^) were observed prior to each approach to avoid sampling dolphins exhibiting evasive behaviors (e.g., deep dives, frequent changes in direction) or groups with neonates (i.e., less than 1 year old) present.

A crossbow device (Barnett Panzer V, 68 kg draw weight, Barnett Outdoors, LLC, Tarpon Springs, FL USA) was used to collect dolphin blubber samples. All biopsy instruments were sanitized following a standardized protocol.^55^ Darts fitted with a 10 × 25 mm stainless-steel sampling tip (Ceta-Dart, Copenhagen, Denmark) were aimed 0.07–0.1 m below the dorsal fin and deployed from a distance of 3–7 m. Externally beveled edges and internal angled prongs in the sampling tip held the tissue in place. Foam affixed to the anterior end of the dart enabled the dart to immediately eject upon penetration and the sample to float above the water. Biopsied tissue was immediately processed on ice by using a cutting board covered with a sterile Teflon sheet. The skin layer was removed from the blubber and stored separately for genetic sexing. Blubber tissue was used for hormone detection and quantification. Skin and blubber samples were flash-frozen in a liquid nitrogen vapor shipper until they were returned to laboratory facilities and transferred to a −80 °C freezer.

Photographs of dolphin dorsal fins for subsequent identification (based on distinct dorsal fin markings^56^) and of each biopsy attempt were collected to prevent resampling of individuals using a Sony Cyber-Shot RX10 IV (2022) or Canon EOS 60D (2012–2014) camera. Data on water parameters (salinity and temperature, measured using a YSI Pro Solo, Yellow Springs, OH USA) (Supporting Information Table S1), group composition (number of dolphins, age classes, predominant behavioral states), and biopsy sample metadata (number of tissue subsamples, location of dart contact with dolphin, individual and group behavior postbiopsy) were recorded. Each dolphin group was monitored for strong behavioral responses to the biopsy dart deployment (e.g., breach and tail slap), and a maximum of three attempts were made to biopsy any group.

### Recovery and Precision Assessment

Using combined techniques,^57,58^ two extraction solvents (acetonitrile and Milli-Q water) and three blubber tissue masses (50, 150, and 400 mg) were tested to develop the hormone extraction protocol for this study. A stranded fresh postmortem bottlenose dolphin was used to obtain large homogeneous sections of blubber for replicate analyses. The lipid content of fresh postmortem stranded dolphins is 44.58%, which is comparable to live free-ranging dolphins and accepted for evaluating blubber matrix effects and hormone extraction efficiency.^57^ Replicates of blubber tissue masses in acetonitrile and Milli-Q water were spiked with isotopically labeled internal standards and compared to a standard calibration curve. Reproducibility was assessed by calculating recovery (% hormone extracted) and precision (% relative standard deviation) values in replicates of each mass and extraction solvent (Supporting Information Table S2).

### Internal and Calibration Standards

**Figure 2:**
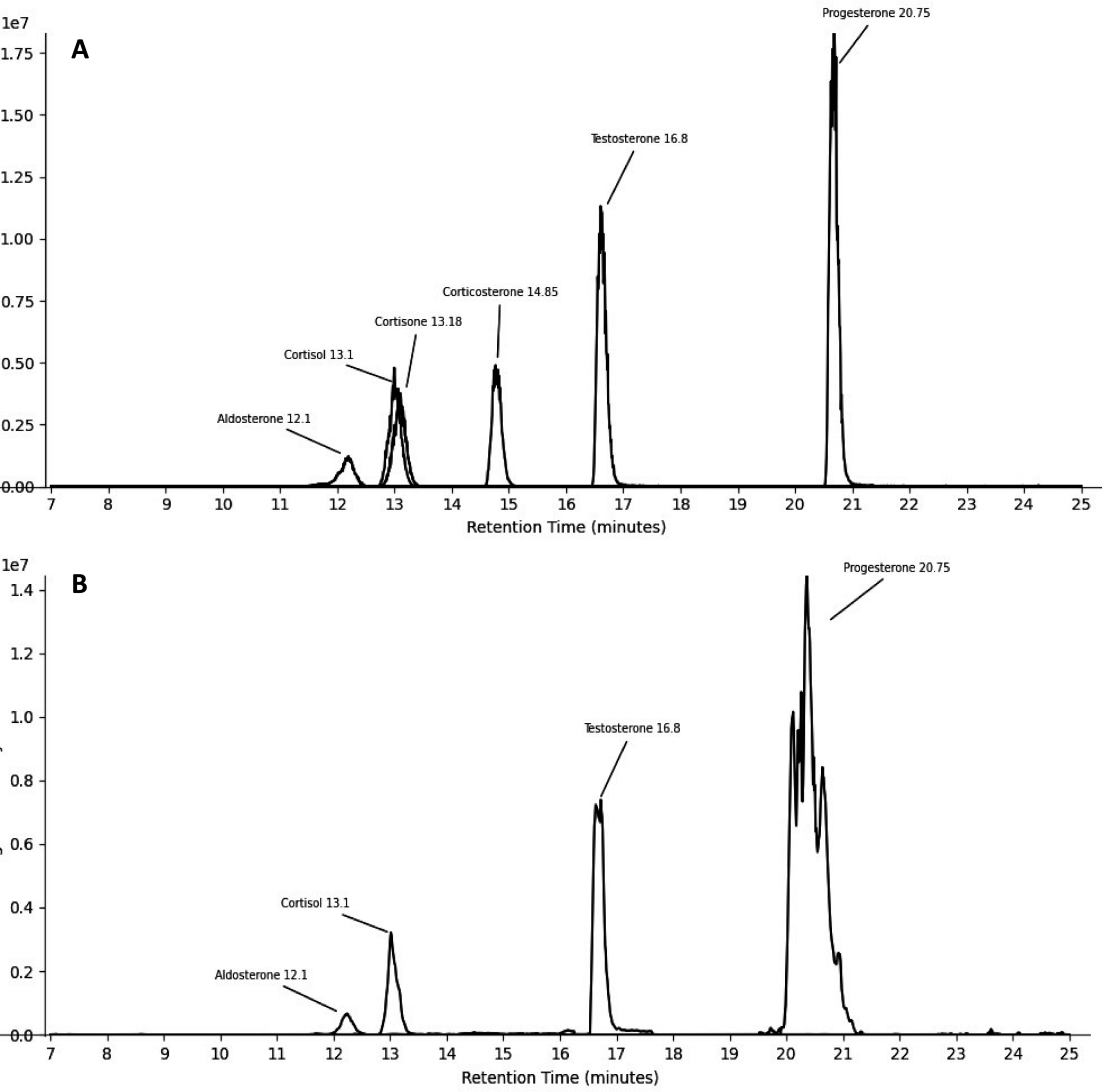
Extracted ion chromatograms of nonlabeled (A) and isotopically labeled (B) hormone standards with associated steroid names and retention times.

Isotopically labeled internal standards (aldosterone-^13^C_3_, cortisol-9,11,12,12-D_4_, progesterone-2,3,4-^13^C_3_, and testosterone-2,3,4-^13^C_3_; Certilliant, Round Rock, TX) and nonlabeled standards (aldosterone, corticosterone, cortisol, cortisone, progesterone, and testosterone; Certilliant, Round Rock, TX) were determined to have ≥98% purity by the manufacturer (Supporting Information Table S3). Since labeled standards are not manufactured for cortisone and corticosterone, aldosterone and cortisol, respectively, were used as surrogate standards due to structural similarities. A distinct mass fragmentation database was created for each individually targeted steroid to ensure analyte separation and confirm analyte detection (Figure 2).

### Blubber Homogenization and Hormone Extraction

Based on recovery and precision analyses (Supporting Information Table S2), stress hormones (aldosterone, corticosterone, cortisol, and cortisone) and reproductive hormones (progesterone and testosterone) were extracted from blubber tissues. Frozen blubber samples were thawed on ice, and 150 mg of tissue was excised from each sample. Blubber tissues were minced on a sterilized glass Petri dish on dry ice and added to homogenization tubes prefilled with 1.4 mm zirconium beads (OPS Diagnostics; PFMB 1400-100-32). An aliquot of 1200 μL of acetonitrile (LC-MS optima grade, Fisher Scientific) was added to each sample tube. Samples were homogenized (Bertin Precellys Evolution homogenizer) three times for 30 s at 6,500 rpm with 5 min intervals on ice to maintain a stable temperature. Samples were centrifuged (VWR 2405-37) at 4 °C for 5 min at 7300 rpm and stored overnight at −20 °C.

After at least 12 h, the supernatants were transferred into glass test tubes. An aliquot of 800 μL of acetonitrile was added to the original homogenization tubes, vortexed for 30 s, and centrifuged for 5 min at 4 °C and 7300 rpm to rinse the tubes. The supernatants were extracted and added to the glass test tubes, and the original homogenization tubes were rinsed twice more. The supernatants collected in the glass test tubes were vortexed for 30 s. An aliquot of 2.75 mL of hexane was added to remove the fat (but not the hormones) from the acetonitrile layer. After vortexing the solution for 30 s to separate layers and waiting at least 10 min, the hexane layer (top layer) was removed and the hexane rinse was repeated until the fat layer was removed. The samples were stored at −20 °C overnight.

The samples in the glass test tubes were dried at 30 °C (Labconco CentriVap) and reconstituted in 500 μL of methanol (LC-MS optima grade, Fisher Scientific) to remove traces of the lysing matrix (debris from homogenization beads). After vortexing for 30 s, the samples were sonicated for 5 min at 20 °C, transferred to centrifuge tubes with a membrane-containing filter (Corning Costar Spin-X), and centrifuged at 4 °C and 7300 rpm. The filtered samples were transferred to new sterile glass tubes, and the original tubes were rinsed with 500 μL of methanol and vortexed for 30 s to extract additional supernatants. The samples were dried, reconstituted in 150 μL of acetonitrile, and transferred to autosampler vials. All samples were stored at −20 °C until instrumental analysis.

### Instrumental Analysis

A 1.7 μm ACQUITY UPLC BEH C18 reversed-phase column by Waters (130 Å, 1.7 μm, 2.1 × 150 mm) was used on a Thermofisher Vanquish ultrahigh-performance liquid chromatography (UHPLC) system coupled with an Orbitrap Fusion mass spectrometer to analyze steroid hormones. Reverse phase chromatographic separation was achieved by using a programmed gradient with the following mobile phases: Eluent A (Milli-Q) with 0.1% (v/v) formic acid and Eluent B (acetonitrile) with 0.1% (v/v) formic acid were mixed with curve 5 to a flow rate of 0.200 mL/min. The total run lasted 31 min with a 7 min re-equilibration and the following gradient: 0–2 min hold at 5% B, ramp to 65% B for 18 min, ramp to 100% B for 1 min, and hold at 100% B for 3 min. The injection volume was adjusted to 5 μL to improve the chromatographic peak shapes. The heated electrospray ionization (H-ESI) was set to 3,500 V for positive spray voltage with the ion transfer tube temperature at 300 °C and vaporization temperature at 225 °C. The three gases on the H-ESI were 35 arbitrary units (a.u.) for sheath gas, 7 a.u. for auxiliary gas, and 0 a.u. for sweep gas. The Orbitrap was run at 120,000 (fwhm at mass-to-charge ratio (*m*/*z*) 200) resolution and mass range of 85–700 *m*/*z* with a S-lens radio frequency (RF) level at 40%. Following the full scan, two fragmentation mass spectra (MS^2^) were acquired with the ion trap via two filters, dynamic exclusion (*n* = 3 for 60 s) and intensity threshold (minimum = 1000). Both MS^2^ scans were isolated with the Quadrupole (0.7 *m*/*z* isolation window), with one fragmentation scan generated through collision-induced dissociation (CID) with assisted energy collision and the other fragmentation scan generated through higher energy collisional dissociation (HCD) with stepped energy collision. The MS^2^ scan with CID had an automatic gain control (AGC) set at 3.0 × 10^4^ and a maximum injection time of 50 ms. The MS^2^ scan with HCD had an AGC of 1.0 × 10^4^ and a maximum injection time of 50 ms. Labeled proline-^13^C_5_,^15^N (Sigma-Aldrich) was used as the internal locking mass standard, while labeled valine-^13^C_5_,^15^N (Sigma-Aldrich) was used to evaluate the mass locking during the entire retention time. The internal standards for the on-the-fly calibration were added in a solution of 96.7% CH_3_CN, 3% H_2_O, and 0.3% HCOOH. The locking solution was introduced to the sample using a T-shaped connection after separation and before H-ESI using an external metering pump (Dionex AXP-MS) at a flow rate of 0.05 mL/min.^59^

Skyline (MacCoss Lab, University of Washington) and TraceFinder 5.1 (Thermo Scientific) were used to process and analyze data. Analytes were identified based on the retention time of the isotopically labeled standard and the compound’s *m*/*z*; the mass error (<2 ppm) assigned to each signal peak was assessed to confirm analyte detection, and MS^2^ was set to 700 mmu. Reference spectra were compiled with external standards at concentrations between 10 and 250 ppb with Compound Discoverer 3.2 (Thermo Scientific). Peak detection and areas were calculated using the Gensis algorithm with a signal-to-noise (S/N) threshold of 3 and a minimum of three fragments necessary for MS^2^ matching. The limit of detection (LOD) was estimated via 9 replicate injections of the target hormones at the lowest concentration yielding a S/N > 3. The limit of quantification (LOQ) was estimated via 9 replicate injections yielding a S/N > 10; if the value was the same as the LOD, the next lowest concentration was reported. Concentrations were corrected for potential loss during hormone extraction using the percent recovery of each analyte. The final concentration (ng/g) was determined by dividing the corrected values by the amount of blubber used (g).

### Genetic Sex Determination

The genetic sex of dolphins sampled in RB between 2012 and 2014 and in MS in 2013 was provided by the NOAA Southeast Fisheries Science Center Marine Mammal Molecular Genetics lab. Sex determination of samples collected in 2022 was performed following a standardized protocol^60^ using the skin layer of the tissue (Supporting Information Text S1). Regions of the X and Y chromosomes were amplified by targeting the ZFX and SRY genes, respectively (Supporting Information Table S4).

### Statistical Analyses

Statistical analyses were conducted in R software (v.4.1.2). Dolphin sex was determined for all samples. Based on LOD and LOQ values, not all hormones were detected nor quantified in each sample. Quantifiable hormone data were tested for assumptions of normality and homogeneity of variance. A Pearson’s *R* correlation and logistic regression analyses were used to assess the relationship between hormone concentration and predictor variables (water temperature, salinity, dolphin sex, and season). Multicollinearity between predictor variables was assessed using the variance inflation factor (VIF) prior to regression analyses to prevent artificially inflating the significance of predictors in the model. A Kendall’s Tau correlation test was used to explore the relationships between hormone concentration, salinity, and water temperature when a hormone was quantified in <10 samples. A Kruskall–Wallis test was used to assess dolphin hormone concentrations relative to season. A Mann–Whitney *U* test was used to assess dolphin hormone concentrations relative to sex when a hormone was quantified in both females and males. Aldosterone and cortisol concentrations from RB samples were compared between 2012–2014 and 2022 using an unpaired two-sample *t* test.

## Results

A total of 82 blubber samples were collected opportunistically across seasons and years from different dolphins, of which 52 samples were collected 10 or more years ago (Table 1). Of the 52 historic samples, hormones were quantified in 22. Approximately equal numbers of samples were collected in the MS between winter and summer and in the ULM between spring and fall (Table 1). In RB, more samples were collected in spring and summer than in the fall. Male bias in sampling was more prominent for RB and ULM, while more females than males were sampled in MS. Regression models indicated variance inflation when season was included; no collinearity concerns were observed between water temperature, salinity, and dolphin sex when season was removed from the model (all VIF values ≤1.5).

**Table 1:** Survey Efforts and Biopsy Sample Metadata

site	season (month)	year(s)	days surveyed	survey area (km^2^)	unique dolphins sighted	unique dolphins sampled (female:male)
MS	winter (January)	2013	6	643	95	15 (10:5)
	summer (August)	2013	6	643	200	15 (8:7)
RB	summer (June)	2012	11	56	198	14 (5:9)
	summer (June)	2013	1	56	51	5 (1:4)
	summer (June)	2014	2	56	36	3 (1:2)
	spring (May)	2022	7	56	144	11 (2:9)
	fall (November)	2022	4	56	85	7 (0:7)
ULM	spring (May)	2022	3	46	74	7 (2:5)
	fall (November)	2022	2	46	29	5 (1:4)

Data include sampling site (Mississippi Sound, MS; Redfish Bay, RB, Upper Laguna Madre, ULM), season (month), year(s), number of days surveyed, size of survey area, number of unique dolphins sighted per season, number of samples obtained, and ratio of females to males sampled.

Recovery (%) and precision (%) for all analytes except progesterone were within accepted Environmental Protection Agency limits (Supporting Information Table S4). All hormones except progesterone could be quantifiable in the blubber samples (Table 2).

**Table 2:** Mean Concentrations of Quantifiable Blubber Steroid Hormones^a^

hormone	female			male		
	mean ± SE	range	*N*	mean ± SE	range	*N*
aldosterone	52.0		1	60.6 ± 9.7	35.1–75.9	4
corticosterone	ND			9.6 ± 0.5	8.9–10.7	3
cortisol	10.3 ± 0.4	9.3–11.2	5	10.44 ± 0.3	9.62–11.7	8
cortisone	10.9		1	12.1 ± 1.3	10.8–13.3	2
progesterone	ND			ND		
testosterone	ND			29.6 ± 5.0	5.6–63.5	16

a Concentrations (ng/g of blubber ± relative standard deviation) were measured in female and male bottlenose dolphins. No data (ND) indicate that hormones were not quantified within female or male subgroups.

### Salinity and Water Temperature

**Figure 3:**
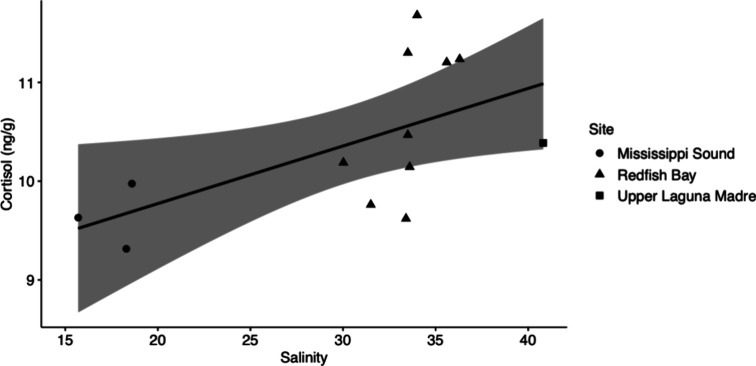
Positive correlation between cortisol (ng/g of blubber) and salinity level. Black line represents the fitted regression line. Shaded region represents the confidence interval. Results of the correlation test are *R* = 0.6 and *p* = 0.03. Shapes denote the three field sites.

Aldosterone was not significantly correlated to salinity (τ = −0.2, *p* = 0.8, *n* = 5) nor water temperature (τ = 0, *p* = 1, *n* = 5), although the highest concentrations of aldosterone (75 ng/g, *n* = 2) were reported in low (6.8) and average (35.7) salinity conditions. Cortisol was significantly related to salinity (*R*^2^_12_ = 0.4, *p* = 0.04) but not water temperature (*p* = 0.7), with a positive correlation between cortisol and salinity identified (*r* = 0.6, *p* = 0.03, *n* = 13) (Figure 3). Aldosterone was not quantified in any of the dolphins with quantifiable cortisol. No significant correlation was identified between corticosterone, cortisone, or testosterone and salinity or water temperature (*p* > 0.05).

### Seasonality and Sex

Cortisol was not significantly different between seasons (*H*_2_ = 2.1, *p* = 0.4) or dolphin sex (*W* = 25, *p* = 0.5). However, cortisol was only quantified in females during the summer (*n* = 5) and in males during the spring (*n* = 6), summer (*n* = 1), and fall (*n* = 1). Quantifiable sample sizes of aldosterone, corticosterone, cortisone, and testosterone for each demographic were too small for statistical comparison by sex. Aldosterone, corticosterone, cortisone, and testosterone were not significantly different between seasons (*p* > 0.05). Aldosterone was detected in males (*n* = 4) and females (*n* = 1) across all seasons. Cortisone was detected in males (*n* = 2) and females (*n* = 1) in the spring, summer, and winter, while corticosterone was only detected in males (*n* = 3) during the spring and summer. Testosterone was highest in the fall (mean 46.2 ng/g, *n* = 3) and lowest in the spring (mean 21.2 ng/g, *n* = 8) and was only detected in males. Seven male dolphins had detectable levels of testosterone in the spring and summer that fell below LOQ.

### Stress Hormones Over Time

Aldosterone was quantified in two RB dolphins between the 2012–2014 and 2022 survey periods and was higher in 2012 (75.5 ng/g, *n* = 1) than in 2022 (35.1 ng/g, *n* = 1). There was not a significant difference in cortisol concentrations of dolphins sampled from RB in 2012–2014 compared to those sampled in 2022 (*t* = 1.5, *p* = 0.2, *n* = 7).

## Discussion

Bottlenose dolphins are becoming increasingly subjected to variability in their environment, as climate change intensifies extreme weather events. The documented effects of extreme weather on dolphins inhabiting the GoM suggest that chronic physiological stress may be occurring.^61−63^ This study is the first to assess the concentration of stress and reproductive hormones in dolphins across an extreme salinity gradient that includes hypersaline levels and the first to quantify aldosterone in dolphin blubber. Cortisol levels in dolphins increased with salinity, and the highest aldosterone concentration was in extremely low ambient salinity. Understanding the impacts of environmental change on bioindicator species such as bottlenose dolphins can aid in the sustainable management of at-risk dolphin stocks and identify concerns of ecosystem health.

Inherent adaptations may be present in the sampled dolphin stocks based on the salinity conditions of each bay system. One primary mechanism for adapting to variable salinities is the hormonal regulation of sodium and chloride electrolytes, which is facilitated by aldosterone.^14^ Additionally, dolphins may adapt by shifting their primary habitat use within vertically stratified water columns (e.g., deep channels) to reduce osmotic pressures with depth.^64^ Electrolyte shifts imposed by different salinity conditions may stimulate an adrenocorticoid response, as evidenced by an increase in serum aldosterone in conjunction with cortisol in dolphins exposed to low salinity.^16,17^ However, it is unclear whether heightened aldosterone secretion is a homeostatic mechanism for adaptation to salinity, a sign of physiological stress, or a combination. No dolphins in this study with quantifiable aldosterone had quantifiable cortisol, limiting our ability to address the potential relationship to long-term stress captured in blubber. However, the two highest aldosterone levels (MS, winter 2013; RB, summer 2012) occurred in dolphins recently exposed to extreme environmental fluctuations, suggesting an inherent role in the stress response. Specifically, Hurricane Isaac inundated MS with >10 inch of rain in August 2012,^65^ yielding pronounced low salinities the following winter. Unusually high salinities in RB in 2012 were linked to a La Niña event and the most severe 1 year drought in Texas’ history.^66^ We lack sufficient data to definitively link extreme salinities to enhanced aldosterone production indicative of electrolyte imbalance and disrupted osmoregulatory homeostasis in bottlenose dolphins. However, aldosterone levels quantified outside the period of extreme salinity were highest in the fall and winter. Aldosterone levels may increase with feeding to enhance water retention from ingested fish,^67^ and bottlenose dolphin foraging is prominent in the winter as prey migrate.^68−70^ The ecological contexts of the sampled dolphin stocks are important considerations.

Hormone levels in blubber reflect accumulated concentrations over time,^31^ enhancing the retrospective assessment of endocrine function in regulating homeostasis and stress response in complex natural environments. Unlike other matrices such as blood, remote blubber biopsies do not require animal handling and therefore do not reflect immediate stress from sampling,^31,71^ making blubber an ideal matrix for assessing long-term stress in wild dolphins. The significant association between cortisol levels in the serum and blubber of bottlenose dolphins^31,72^ and striped dolphins (*Stenella coeruleoalba*)^73^ suggests that remote blubber biopsies are suitable biomarkers of stress in these dolphin species.^27,31,71−74^ Since a certified reference material for dolphin blubber does not presently exist, it is recommended that study-specific recovery and precision analyses as presented herein be used to evaluate matrix effects rather than a standard reference material from a different species due to variations in matrix composition.^58^

As this is the first study to quantify aldosterone in dolphin blubber, baseline levels have not yet been determined; however, our reported concentrations of aldosterone in blubber are similar to serum levels in free-ranging bottlenose dolphins^21^ and in the blubber of gray whales (*Eschrichtius robustus*).^58^ Low concentrations of aldosterone in bottlenose dolphin tissues could potentially be circumvented through the development of extraction protocols that are more compound-specific.^75^ Because aldosterone has a low affinity (<20%) for binding to albumin and other proteins,^31^ the capacity for aldosterone to bind to unknown proteins warrants further exploration. Extraction from the skin and blubber layers of the same sample could reveal differences in hormone deposition and concentration ratios between different tissues.^38^

Like low salinity, high saline conditions may challenge the homeostatic mechanisms required to maintain osmotic balance in dolphins and induce physiological stress. Dolphin skin is a major conduit for the ingress of water,^76^ with water loss reported in hyperosmotic conditions.^77^ Dolphins may also experience mild dehydration, which can increase both blood and urine osmolality.^78,79^ Cortisol was positively correlated to salinity in this study, and the highest recorded ambient salinity level (40.81 in ULM) corresponded to a dolphin with a high cortisol concentration (10.39 ng/g). Most of the quantifiable cortisol levels were obtained from dolphins sampled during 2012–2014 in RB following the La Niña event and severe 2011 drought, suggesting that high salinity imposes physiological pressures that may elevate dolphin stress. As cortisol was not quantified in samples collected below 15, we could not verify that extremely low saline conditions induced physiological stress.

Both endogenous and exogenous influences may contribute to the positive correlation between salinity and cortisol, such as changes in ambient water temperatures, prey distribution, underlying physiological illness, or the interaction of multiple stressors. Spring and summer are the predominant mating and calving seasons for dolphins in MS^80^ and RB^66^ and are the seasons in which cortisol was quantified the most. Elevated cortisol levels during the mating and calving season are expected due to reduced foraging activities.^81,82^ Reproductive pressures likely account for the detection of cortisone and corticosterone in males during the spring and summer as these hormones are known to correlate with cortisol in dolphins^22,23^ and cortisone–cortisol conversion actively occurs in dolphin blubber.^83^ The interconversion of cortisone and cortisol may also explain the presence of cortisone in one female during the winter, as cortisol peaks during late gestation in female dolphins and parturition generally occurs in the spring.^84^ The difference in the mean cortisol levels (0.14 ng/g) reported for female and male dolphins in this study is minor. Cortisol, cortisone, and corticosterone were not significantly related to season nor quantified adequately in all seasons to draw strong conclusions. Cortisol was not detected in dolphins during seasons with the coldest water temperatures (i.e., MS in winter 2013; average 13.1 °C) when it is expected to be the highest.^18^ Blubber cortisol concentrations reported here were similar to other free-ranging biopsied dolphins^23^ and were nearly 10-fold higher than in managed care dolphins,^31^ suggesting heightened stress in natural environments.^85^ Like aldosterone, baseline blubber cortisol levels have not been established for free-ranging bottlenose dolphins and are warranted across seasons and demographics.

Only two male dolphins had quantifiable aldosterone or cortisol, and testosterone levels were not elevated in conjunction with the stress hormones. The range of testosterone levels in male dolphins of this study is similar to those reported for short-beaked common dolphins (*Delphinus delphis*)^86^ and free-ranging bottlenose dolphins using blubber biopsies.^87^ High testosterone levels prior to seasonal breeding help stimulate the spermatogenic cycle, which is often slow and complex.^88,89^ Testosterone peaked in MS and RB stocks approximately 3 months prior to the known mating seasons, supporting the idea that the increased fall and winter androgen secretion in males may be in preparation for mating in the spring. Due to the poor recovery of progesterone in the blubber, we were unable to determine if a relationship existed with testosterone. While progesterone is predominantly a female pregnancy hormone, it may also precede testosterone synthesis in males through conversion to 17-hydroxyprogesterone.^23^ Testosterone and progesterone levels should be monitored in free-ranging dolphins to better understand their syntheses relative to reproductive events.

The effects of measurable variables (i.e., water temperature, salinity, season, and sex) were isolated in regression analyses based on the level of significance. The high proportion of males biopsied relative to females (65:35%) suggests a strong male bias, consistent with other findings.^90,91^ Potential drivers of sex bias during remote biopsy sampling may include geographic location, individual behavior, nutritional requirements, reproductive condition, and season.^92^ The observed male bias in this study likely reflects efforts to avoid sampling groups with neonates (and their mothers). In addition to sampling bias, there was a higher rate of hormone quantification in samples obtained from males compared to females. Even during pregnancy, blubber progesterone concentrations in female bottlenose dolphins are 6-fold lower than in other dolphin species,^92^ suggesting that quantification of sex steroid hormones in females may be seasonally dependent. Increased sampling of free-ranging female dolphins is needed to better understand the influence of sex, season, water temperature, and salinity on female sex hormones.

Integrating archived and newly collected blubber tissues of dolphins from RB provided a retrospective analysis of how changes over time may influence dolphin health and reflect ecosystem quality. Opportunistic and unequal sampling efforts across time for each system may introduce confounding variables. It is also possible that specific individuals had unique underlying physiological conditions not generalizable to the population and that other extrinsic variables could influence hormones. For instance, following a historic drought in 2011, a multimillion dollar dredging project began in 2015 to broaden and deepen the shipping channel adjacent to RB to accommodate larger vessels and trade operations.^93^ Channel dredging can lead to the resuspension of contaminants in the water column that can bioaccumulate in dolphin tissue and precede or heighten endocrine disruption.^29^ Our data show that cortisol concentrations quantified in archived samples from RB were consistent with those collected in 2022, indicating that dolphins may have adapted to the increasing development and port activity in the area or that the impacts of dredging were not fully captured in the samples collected in 2022. Consistency in cortisol concentrations between 2012 and 2022 also suggests minimal blubber hormone degradation, supporting that prolonged storage time has a minimal effect on blubber hormone concentrations when high sample quality is preserved^94^ and supporting the use of historic blubber for long-term and retrospective studies. Aldosterone was quantified in one 2012 RB sample during an extreme salinity event and one 2022 RB sample when salinity had resumed normal levels, suggesting that elevated aldosterone in 2012 may be linked to extreme salinity and not anthropogenic development. It remains crucial to understand how large-scale climate changes may compound the effects of anthropogenic disturbances in coastal urbanized and industrialized areas that dolphins inhabit. The historic presence of pesticides and heavy metals like zinc and mercury in RB from industrial operations, superfund sites, and dredging raises concerns about their continued persistence and bioavailability in the ecosystem.^45^ Research on hormones and pollutants in biopsied blubber is crucial for monitoring the health of at-risk BSE dolphin stocks.

Like RB, ULM has a history of chemical and heavy metal pollution from agricultural practices that may expose dolphins to trace levels of contamination through consumption of fish.^95^ Additionally, 15 months prior to sampling in ULM, Winter Storm Uri hit Texas with frigid temperatures that led to approximately 3.8 million fish kills along the Texas Coast,^96^ including dominant prey species for dolphins. Most fish communities impacted by historical freeze events in Southern Texas take about 2 to 3 years to recover,^97^ potentially introducing stress to dolphins in ULM reflected in cortisol levels. Following the Deepwater Horizon oil spill in 2010 and Hurricane Isaac in 2012, MS dolphins were documented with severe adrenal and lung diseases,^44^ suggesting that underlying physiological condition may have impacted the hormone profiles of dolphins sampled in 2013. The use of additional health biomarkers and analytical tools could provide more in-depth perspectives on the effect of these events on the overall dolphin health.

The quantification of aldosterone in dolphin blubber marks a pivotal step in advancing the understanding of how dolphins regulate physiological homeostasis across highly variable environmental contexts. The positive correlation between cortisol and salinity suggests a disruption of the osmotic balance and a subsequent need for increased aldosterone production across a salinity gradient. The simultaneous assessment of reproductive hormones in this study provides the additional context necessary for interpreting the level and source of stress in free-ranging dolphins. Hormone concentrations determined from remotely biopsied blubber samples can be informative of reproductive events and large-scale environmental change and are further warranted to monitor the health of free-ranging dolphins and the ecosystems they inhabit.

## Supporting Information Available

Protocol and primer sequences used for determining the genetic sex of dolphins, salinity and water temperature data, comparison of analyte extraction efficiency, and analytical parameters for internally labeled and nonlabeled hormone standards (PDF)

## Abbreviations

BSE: bay, sound, and estuarine
GoM: Gulf of Mexico
MS: Mississippi Sound
RB: Redfish Bay
ULM: Upper Laguna Madre
NOAA: National Oceanic and Atmospheric Administration
UHPLC: ultrahigh-performance liquid chromatography
H-ESI: heated electrospray ionization
a.u.: arbitrary units
*m*/*z*: mass-to-charge ratio
RF: radio frequency
MS^2^: fragmentation mass spectra
CID: collision-induced dissociation
HCD: higher energy collisional dissociation
AGC: automatic gain control
S/N: signal-to-noise ratio
LOD: limit of detection
LOQ: limit of quantification
VIF: variance inflation factor
