# Supplementary material for: Stress and Reproductive Hormones of Free-Ranging Dolphins Across a Natural Salinity Gradient
Source: ACS Omega. 2024 Oct 29;9(45):45068–79. doi: 10.1021/acsomega.4c05466 (PMC11561764; doi:10.1021/acsomega.4c05466)
Supplement: Supplementary file 1 — ao4c05466_si_001.pdf [file ao4c05466_si_001.pdf]

Stress and reproductive hormones of free-ranging dolphins across a natural salinity gradient

Makayla A. Guinn[1*], Justin Y. Elliott[1], Christiana S. Wittmaack[2], Carrie Sinclair[3], Hussain A. Abdulla[1], Dara N. Orbach[1]

[1]Texas A&M University-Corpus Christi, 6300 Ocean Drive, Corpus Christi, Texas 78412, United States

[2]Hydrosphere, 2034 Beacon Avenue, Monroe, North Carolina 28110, United States

[3]National Oceanic and Atmospheric Administration, Southeast Fisheries Science Center, Mississippi Laboratories, 3209 Frederic Street, Pascagoula, Mississippi 39567, United States

Text S1. Protocol for determining genetic sex of dolphins using the biopsied skin layer.

Aliquots of 20 µL of lysis buffer (2.25 mM MgCl2, 15 mM Tris pH 8.3, 75 mM Kcl, 0.0015% Gelatin, 0.3% Tween20, 0.3% NP-40) and 2 µL of proteinaseK (200 ug/mL) were pipetted into a PCR tube with approximately 0.05 mg of dolphin skin sample. DNA from known female and male post-mortem dolphins were used as positive controls. Samples were incubated at 55 °C for approximately 1 hour and then heated for 10 minutes at 95 °C in a thermocycler (Bio-Rad C1000) to deactivate the proteinaseK. A 2 µL aliquot of the lysis mixture was added to 23 µL of the PCR reaction mix (10X PCR buffer (2.5 µL), 0.3 µM of primers ZFX0582F (0.75 µL), ZFX0923R (0.75 µL), PMSRYF (0.75 µL) and 0.06 µM of TtSRYR (0.15 µL), 1.5 mM MgCl2 (included in 10X buffer), 150 µM dNTPs (0.375 µL), 1.5 U Taq DNA polymerase (0.3 µL), and water (17.425 µL). The unidentified skin samples and positive and negative controls were run in the thermocycler at the following program: 92 °C for 30 seconds followed by 35 cycles of 94 °C for 30 seconds, 51 °C for 45 seconds, and 72 °C for 45 seconds. An aliquot of 3.5 µL of DNA was diluted in Milli-Q water to a 10 µL volume and run at 75 V on a 2.5% agarose gel. Fragment sizes produced by the ZFX and SRY primers were approximately 382 and 339, respectively. The presence of one band (female) or two bands (male) was used for sex determination.

Table S1. Salinity and water temperature (°C) data.

Site	Season (Month)	Year(s)	Salinity (Range)	Water Temperature (Range, °C)
Mississippi Sound	Winter (January)	2013	13.7 ± 0.7 (5.9 – 22.3)	13.3 ± 0.1 (12 – 15.8)
	Summer (August)	2013	18.4 ± 0.2 (15.5 – 22.8)	29.1 ± 0.1 (27.4 – 30.8)
Redfish Bay	Summer (June)	2012	34.9 ± 0.2 (30.3 – 37. 9)	30.1 ± 0.1 (28.9 – 39.1)
	Summer (June)	2013	31.1 ± 0.4 (29.3 – 33.5)	30 ± 0.1 (29.2 – 30.4)

	Summer (June)	2014	33.8 ± 0.1 (33.3 – 34)	29.3 ± 0.1 (28.8 – 29.7)
	Spring (May)	2022	30.2 ± 0.1 (28 – 32.2)	27.5 ± 0.2 (21.1 – 29.9)
	Fall (November)	2022	32.2 ± 0.1 (30.2 – 32.9)	22.2 ± 0.7 (15.6 – 25.5)
Upper Laguna Madre	Spring (May)	2022	36.7 ± 0.2 (35.3 – 40)	27.4 ± 0.4 (20.3 – 28.8)
	Fall (November)	2022	40.3 ± 0.3 (38.5 – 43.2)	21.9 ± 0.8 (18.5 – 26.1)

Description of salinity and water temperature (°C) data collected for each sampling site, season (month), and year(s). Salinity and water temperature measurements are listed as mean ± standard error. Parenthesis denotes parameter range.

Table S2. Comparison of analyte extraction efficiency.

	Milli-Q Water (n=15)		Acetonitrile (n=9)	
Analyte	Recovery (%)	Precision (%)	Recovery (%)	Precision (%)
Aldosterone				
50 mg	47.98	13.41	61.75*	7.72
150 mg	68.76*	21.71	51	4.82
400 mg	54.78	22.84	52.99	9.16
Cortisol				
50 mg	68.44	20.24	87.54*	8.68
150 mg	99.23*	31.72	74.54	7.64
400 mg	75.17	35.73	75.24	9.77
Testosterone				
50 mg	27.10	4.07	83.23*	11.63
150 mg	24.38	9.10	70.96*	2.22
400 mg	11.43	2.58	86.42*	17.67

Progesterone				
50 mg	0.18	0.04	42.65*	4.93
150 mg	0.33	0.02	36.18*	2.63
400 mg	1.04	0.03	39.90*	15.05

Asterisks (*) indicate a significantly higher extraction in the respective solvent ($p < 0.05$). High recovery (%) and precision (%) are considered more efficient at extracting analytes. Recovery and precision values are based on a 100% scale.

Table S3. Analytical parameters for internally labeled and non-labeled hormone standards.

Analyte	Molecular Formula	Adduct	Precursor Ion (m/z)	Retention Time (min)	LOD (ppb)	LOQ (ppb)
Aldosterone	$C_{21}H_{28}O_5$	$[M + H]^+$	361.2010	11.85	1	2.0
Aldosterone ^{13}C	$^{13}C_3C_{18}H_{28}O_5$	$[M + H]^+$	364.2110	11.85	-	-
Corticosterone	$C_{21}H_{30}O_4$	$[M + H]^+$	347.2217	14.85	0.3	0.5
Cortisol	$C_{21}H_{30}O_5$	$[M + H]^+$	363.2166	13.1	0.3	0.5
Cortisol D_4	$C_{21}H_{26}D_4O_5$	$[M + H]^+$	367.2110	13.1	-	-
Cortisone	$C_{21}H_{28}O_5$	$[M + H]^+$	361.2010	13.18	0.5	0.7
Progesterone	$C_{21}H_{30}O_2$	$[M + H]^+$	315.2319	20.75	0.1	0.3
Progesterone ^{13}C	$^{13}C_3C_{18}H_{30}O_2$	$[M + H]^+$	318.2419	20.75	-	-
Testosterone	$C_{19}H_{28}O_2$	$[M + H]^+$	289.2162	16.8	0.1	0.5
Testosterone ^{13}C	$^{13}C_3C_{16}H_{28}O_2$	$[M + H]^+$	292.2263	16.8	-	-

Molecular formula and retention times of target metabolites. Limit of detection (LOD) and quantification (LOQ) values were used to determine the threshold for detection and quantification of each analyte. One part per billion (ppb) is equivalent to one ng/g.

Table S4. Primer sequences used for sex determination.

Primer	Sequence (5' – 3')	Reference
SRY Gene		

TtSRYR	ACCGGCTTTCCATTCGTGAACG	Rosel (2003)
PMSRYF	CATTGTGTGGTCTCGTGATC	Richard et al. (1994)
ZFX Gene		
ZFX0582F	ATAGGTCTGCAGACTCTTCTA	Berubé and Palsbøll (1996)
ZFX0923R	AGAATATGGCGACTTAGAACG	Berubé and Palsbøll (1996)
